# Open-Cell Aluminum Foams by the Sponge Replication Technique

**DOI:** 10.3390/ma12233840

**Published:** 2019-11-21

**Authors:** Alina Sutygina, Ulf Betke, Michael Scheffler

**Affiliations:** Department of Mechanical Engineering, Institute for Materials and Joining Technology, Otto-von-Guericke-University Magdeburg, Große Steinernetischstraße 6, 39104 Magdeburg, Germany; ulf.betke@ovgu.de (U.B.); m.scheffler@ovgu.de (M.S.)

**Keywords:** aluminum foams, porous materials, sponge replication technique

## Abstract

Open-cell aluminum foams were manufactured by a sponge replication technique having a total porosity of ~90%. The influence of the thermal processing conditions such as atmosphere and temperature on the cellular structure, phase composition porosity, thermal conductivity, and compressive strength of the foams was studied. It was found that the thermal processing of aluminum foams in Ar at temperatures up to 800 °C led to aluminum foams with a reduced strut porosity, a lower amount of aluminum oxide, a higher thermal conductivity, and a higher compression strength, compared to foams thermally processed in air. These results were explained by the lower amount of aluminum oxide after thermal processing of the foams.

## 1. Introduction

Metal foams have received a great deal of attention due to their unique property combinations such as low density, lightweight (porosity can achieve 98%), electrical/thermal conductivity, non-flammability, high stiffness-to-weight ratio, energy/acoustic dissipation, and high bending strength [1,2]. They found widespread applications in the aerospace industry, architecture, automotive industry, filters, heat exchangers, biomedical prostheses, sound barriers, vibration dampers, and as high strength-to-weight ratio materials (syntactic metal foams) [1,2,3,4,5,6,7,8]. Metal foams might be able to substitute a variety of conventional materials. For example, the use of open-cell metal foams as catalyst carries instead of honeycomb support materials increases a range of shapes and sizes of manufactured materials. These substrates show improved mixing of reactants and enhanced surface reactions due to its higher tortuosity [9].

Pure metals and varieties of their alloys are used for the manufacturing of metal foams [10,11,12,13]. Among the various open-cell metal foams with high thermal/electrical conductivity and liquid/gas permeability, aluminum foams have emerged as promising material in areas such as heat exchangers, filters, catalyst carriers, and electrodes in aluminum-air batteries due to low cost, relatively easy manufacturing of required functional geometries, and better mechanical properties compared to other low melting metals [14,15,16,17,18,19]. The most widespread methods to produce open-cell metal foams are investment casting, casting around hollow spheres, metal injection molding, and space holder casting [20,21,22,23]. However, there are some weaknesses as a non-uniform cellular structure which depends on the shape of space holders [24], relatively low specific surface area of foams and high production cost and high complexity of manufacturing processes [21,24,25,26].

One of the promising ways to manufacture open-cell metal foams is the sponge replication technique [27], which was originally applied for ceramic foams [28,29]. This method consists of only three production steps: (1) coating of a polyurethane (PU) template with an aqueous metal powder/binder slurry; (2) thermal removal of the PU templates and binder; (3) final sintering (thermal processing) of the foams. Those metal foams have controllable pore size, a high porosity (>90%) and a high surface-area-to-volume ratio, which make them attractive for a variety of thermal management and catalytic applications [30,31]. The main limitations of the sponge replication technique are the existence of thin oxide shells around sintered powders which are hard to disrupt and high oxidation rates of powders during the sintering step at high temperatures [32,33]. To control the sintering process of metal powders different sintering atmospheres, additions to the powders and different sintering temperatures are applied.

The sponge replication technique has been successfully applied for the manufacturing of open-cell titanium [34,35], copper, and Ti6Al4V foams [30,36]. In ref. [37] the feasibility of processing of aluminum foams as prepared by the sponge replication technique was demonstrated. The relatively stable aluminum foams, thermally treated in air at 620 °C for 4 and 7 h, showed a porosity between 94.4–95.5%. This work was focused on the determination of the slurry composition. Despite the general producibility of stable aluminum foams no further investigations were carried out.

The aim of this work is the manufacturing of open-cell aluminum foams by the sponge replication technique to identify critical parameters of the feasibility of Al foam manufacturing with the replica process with an aqueous aluminum slurry.

## 2. Materials and Methods

### 2.1. Specimen Preparation

For the manufacturing of aluminum foams, an air-atomized aluminum powder, supplied by Ecka Granules (MEP103 RE903, Ranshofen, Austria) with a purity of 99.5 wt.% (titanium <0.25 wt.%) and average particle size <10 µm was used. The aluminum powder was mixed with a 10.7 wt.% solution of a polyvinyl alcohol binder (1.2 wt%, Optapix PA 4G, Zschimmer and Schwarz Chemie GmbH, Lahnstein, Germany) in distilled water. The solid content of Al powder in the final dispersion was 70.6 wt.%. The dispersion was mixed at 2000 rpm for 6 min using a planetary centrifugal mixer (THINKY Mixer ARE-250, THINKY Corp. Tokyo, Japan) and cooled to room temperature since the temperature increased during mixing.

An open-cell PU foam with a linear cell count of 20 ppi and a geometric size of 20 mm × 20 mm × 20 mm (Koepp Schaum GmbH, Oestrich-Winkel, Germany) was used as a template. For the measurement of the thermal conductivity, the geometric template size was 20 mm × 50 mm × 50 mm. The PU foam was dipped into the corresponding dispersion and excess desperation was removed by air blowing. Then the dispersion-coated templates were dried for 24 h at room temperature. The binder and PU burning out were carried out in air at 250 °C for 3 h and at 500 °C for 3 h in a circulating air furnace (KU 40/ 04/A, THERMCONCEPT Dr. Fischer GmbH, Bremen, Germany). The final thermal processing step was carried out in a conventional tube furnace (alumina tube, HTRH 70-600/1800, Carbolite-Gero GmbH and Co. KG, Neuhausen, Germany). The foams were thermally processed for 3 h in air and Ar atmosphere (purity 99.999%) at 750 °C and in Ar at 750, 800, 850, and 900 °C for 3 h. The thermal processing conditions are listed in Table 1. The heating and cooling rates were 3 K/min for both. In the case of Ar as thermal processing atmosphere, the flow rate was 25 mL/min.

### 2.2. Characterisation

A scanning electron microscope (SEM; FEI ESEM XL30 FEG, Hillsboro, OR/USA) was used to characterize the powder morphology and the microstructure of cross sections of the thermally processed foams. The foams were vacuum impregnated in an epoxy resin, grinded (180, 320, 600, 800, 1200, 2500, 4000 mesh grinding paper), and polished (diamond suspension 3 and 1 µm). A Hydro 2000SM (Malvern Instruments, Malvern, United Kingdom) particle size analyzer was used for the measurements of the particle size distribution of the powder.

The thermal transformation with respect to weight change and the oxidation onset of the powder were analyzed with differential scanning calorimetry (DSC) and thermogravimetry (TG) which were conducted simultaneously using a thermal analyzer STA 449 F3 Jupiter (Netzsch-Gerätebau GmbH, Selb, Germany). The powder was heated from room temperature to 800 °C in air with a flow rate of 50 mL min^−1^ and a heating rate of 10 K·min^−1^.

X-ray diffraction analysis (XRD) was conducted with an X’Pert Pro diffractometer (PANalytical GmbH, Kassel, Germany, Co K_α_^1/2^ radiation, 2θ, 40–85°) with Bragg–Brentano geometry. The Rietveld analysis for determination of the phase composition in the supplied powders and the thermally-processed foams was performed with the Topas Academic V5 software package [38].

The total porosity was measured based on the calculation of the geometric foam density and the skeletal density of the strut material (V_pores_/V_foam_) [39] (density of the pure bulk Al ρ_Al_ = 2.7 g·cm^−3^ [40]). The strut porosity (V_strut pores_/V_struts_) of the thermally processed foams was quantified by the Archimedes method using water as an infiltrating fluid according to the DIN EN 623-2:1993-11 standard procedure [41].

According to the DIN standard superficial water adherence to the sample has to be removed. However, due to the complex geometry of the obtained foams, it could not be performed because of residual water within the strut cavities after the PU burnout. Therefore, these cavities are included formally during the measurements into the strut material porosity. This results in a slight overestimation of the strut porosity values. To solve this problem, the volume of the PU foam struts was subtracted from the total strut pore volume. This approach can exclude cavities after the PU burnout from the material porosity of the foam struts [42]. This value was calculated from the average template weight of 1.52 g for a 50 cm^3^ foam piece and a PU skeletal density of 1.1 g·cm^−3^ according to He-pycnometry [42]. The volumes of the cavities after PU burnout were corrected by the volumetric shrinkage of the thermally-processed foams.

Measurements of the thermal conductivity and diffusivity were carried out with the transient plane source (TPS) technique using a TPS 2500 S (Hotdisk SE, Gothenburg, Sweden). The sensor, which was 9.868 mm in diameter, was placed in between two foams sanded previously [43]. The heating power was 200 mW for 5 measurements for 5 s per each measurement. The samples were turned, therefore all sides were measured and a total of 20 measurements were obtained. The sensor temperature changes were used for the calculation of the thermal conductivity [44].

The compression strength was determined with a TIRAtest 2825 testing machine (TIRA GmbH, Schalkau, Germany) with a loading plate of 150 mm in diameter. The applied load was set to 2 mm·min^−1^. For the calculation of the compressive strength, 10 specimens were used for each thermal processing parameter. The calculations of the Weibull modulus and Weibull parameter m for the compressive strength for each sample series were carried out with the software package Visual-XSel 14.0 (CRGRAPH, Starnberg, Germany).

## 3. Results

**Figure 1:**
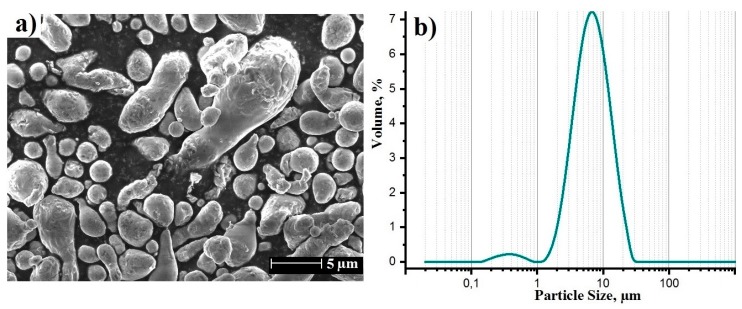
SEM image of the starting powder (**a**) and its particle size distribution (**b**).

Figure 1 represents a SEM image of the aluminum powder and the particle size distribution. The powder had a smooth surface and is of a spheroidal geometry. From the obtained results it follows d_50_ = 6.2 ± 0.3 µm with 10% of all particles <2.7 µm and 10% >13.4 µm.

**Figure 2:**
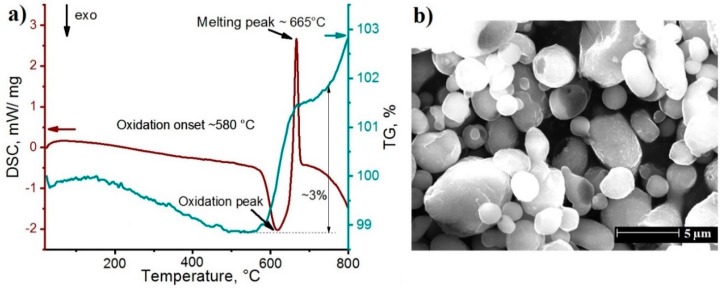
Thermogravimetry (TG) and differential scanning calorimetry (DSC) curves of the powder (**a**) and SEM image of the powder after TG and DSC measurements (**b**).

Figure 2a shows the TG and DSC curves of the aluminum powder. Mass loss was observed below 400 °C which was assigned to the desorption of gaseous species, water and other airborne species absorbed on the powder surface. The powder showed an exothermic oxidation reaction and a subsequent endothermic peak assigned to the melting process. The onset temperature of the oxidation reaction was ~580 °C. There was a continuous weight gain after the start of the oxidation reaction, simultaneously the rate of the weight gain decreased with the onset of the endothermic reaction with the melting peak at T_mAl_ = 665 °C. With further increase of the temperature, a further mass gain of the powder was observed. The total weight gain was ∼3 wt.% at 750 °C.

After heating to 800 °C the powder particles kept their shape and virtually did not melt despite the melting point of the powders as found at 665 °C. This is explained by a thin alumina layer on the powder surface [32,33], which is not disrupted and stabilizes the original particle shape.

**Figure 3:**
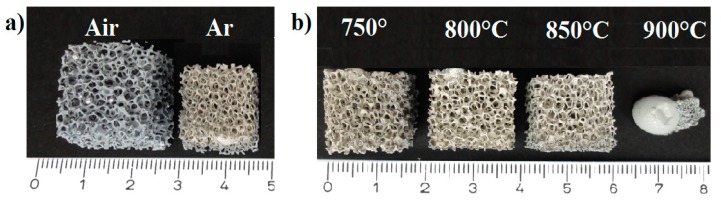
Al foams after thermal processing for 3 h in air and Ar at 750 °C (**a**) and Ar at 750–900 °C (**b**).

The foams after thermal processing in air at 750 °C and Ar at 750–900 °C for 3 h are shown in Figure 3a and b, respectively. After thermal processing in air, there were small aluminum beads on the strut surface of the foam. The foams thermally processed in Ar atmosphere showed a significantly higher shrinkage in all directions. These foams had some larger, formerly molten aluminum beads located in their interior on the strut surfaces. However, there was no significant change of the foam shapes up to 850 °C after thermal processing in Ar. A further increasing of temperature to 900 °C led to the destruction of the foam structure caused by melting of aluminum and subsequent collapse (Figure 3b).

**Figure 4:**
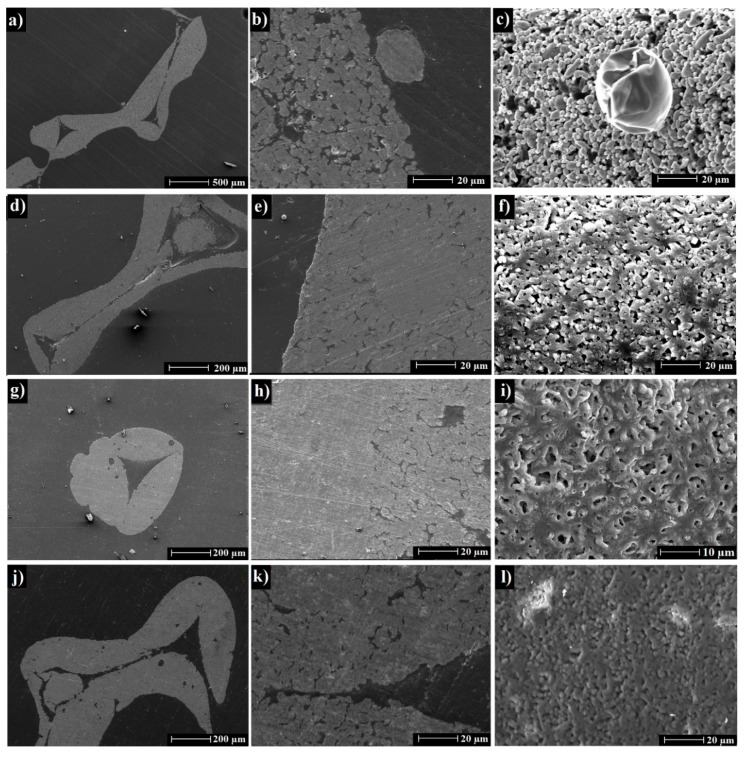
SEM images of aluminum foams thermally processed for 3 h at 750 °C in air (**a**–**c**) and Ar at 750 °C (**d**–**f**), 800 °C (**g**–**i**), and 850 °C (**j**–**l**).

Figure 4 shows SEM images of cross-sections and surface morphology of Al foams thermally processed at 750 °C in air and Ar at 750–850 °C. The struts were hollow which originated from the manufacturing process. The cavities in struts were the result of the replication process of the PU foams. Some formerly molten aluminum beads were located outside and inside the foam struts.

The microstructure of the foam struts appeared inhomogeneous with pores in the strut walls resulting from incomplete thermal processing. This may have been caused by the presence of a thin alumina layer on the powder surface, which is typical for aluminum powders even if they are coated with a protection layer [32,33]. As seen in Figure 4d–f, the thermal processing in Ar led to samples with less porosity and less voids within the struts compared to samples thermally processed in air. Those strut walls were almost dense with less porosity, (Figure 4e). It was seen from the comparison of the surface morphology (Figure 4c,f) that the powder particles kept their shapes after thermal processing in air in contrast to the thermal processing in Ar where the powder particles were more merged and densely packed.

From the SEM images of foams thermally processed for 3 h in Ar at 750–850 °C (Figure 4d–l) it was observed, that the increase of the temperature improved the powder particles merging. The pores within the struts still existed; however, the powder particles formed agglomerations of melt powders with fewer pores in the struts with the increase of the thermal processing temperature. However, there was an obvious deformation of the strut shape/strut structure at 850 °C.

**Figure 5:**
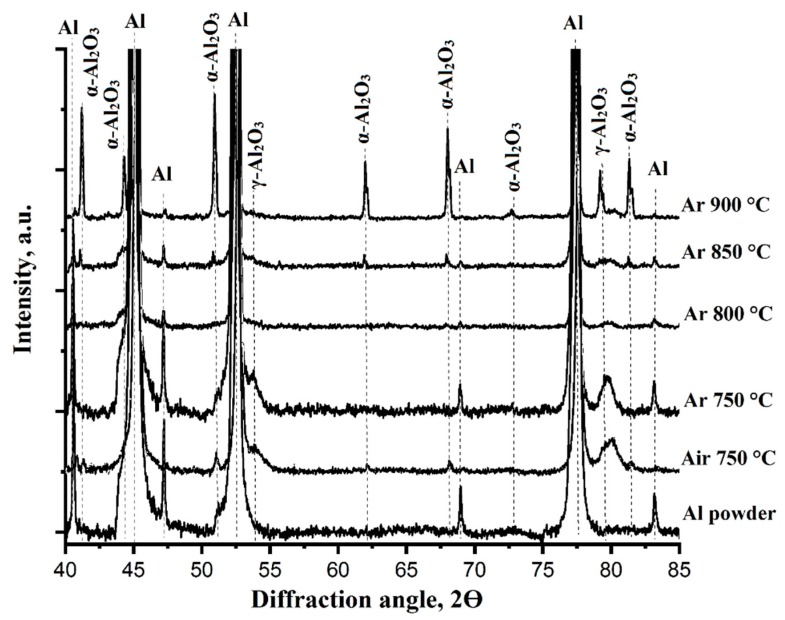
X-ray diffraction patterns of the as-received powder and aluminum foams after thermal processing in air and Ar at 750 °C and in Ar at 750–900 °C.

The results of the XRD phase analyses indicate the formation of aluminum oxides (α-, γ-Al_2_O_3_) after thermal processing of the Al foams (Figure 5). For the phase analyses, the samples were compressed (except the sample thermally processed at 900 °C) after thermal processing in Ar in order to obtain a planar surface for proper measurement. The foams thermally processed in air were milled and the resulting powder was analyzed.

The starting aluminum powder as-received from the supplier consisted of an aluminum phase; thin alumina layer was not detected by XRD analysis, probably because this amount of alumina was below the error of the XRD measurement or it could also have been in amorphous state [32,33]. The highest amount of aluminum oxide after thermal processing in air at 750 °C was ~20.2 wt.% of α-Al_2_O_3_ and ~2.4 wt.% of γ-Al_2_O_3_. The foam collapsed after thermal processing in Ar at 900 °C showed ~28.8 wt.% of α-Al_2_O_3_ and ~7.4 wt.% of γ-Al_2_O_3_; however, due to inadequate sample preparation from the drop-like bulk material, formerly molten (see Figure 3b), this composition was not representative and will not be discussed any further. Most likely, the residual foam structure as seen in Fig. 3b on the right consisted mainly on the oxide shells of the former aluminum powder particles.

The phase compositions of the as-received powder and the aluminum foams thermally processed for 3 h at 750 °C in air and at 750–900 °C in Ar from XRD measurements and calculated with Rietveld analysis are listed in Table 2. From these results, the formation of aluminum oxides was evident. While after processing in air the alumina amount is high—22.6 wt.%—the samples processed in Ar showed moderate (3.8 wt.% to 5.8 wt.%) alumina amounts; in the case of the sample processed at 900 °C the alumina amount was ~36.2 wt.%. This might be explained by the collapse of the foam structure and the redistribution of the two phases: the samples were not homogenized prior to XRD analysis. However, it is not intended in this paper to address the alumina formation; it is to demonstrate the feasibility of Al foam processing with the Schwartzwalder process. To reduce the alumina content during processing will be the aim of the forthcoming work.

From the phase analysis results the following conclusions may be drawn: The foams thermally processed in Ar have thinner oxide shells around the particles in comparison with the foams thermally processed in air. It is known that the disruption and dissolution of the oxide layer is one of the key problems of aluminum powders sintering and thermal processing [45,46]. This is connected to the melting point of aluminum oxide which is ~2072 °C [47]. However, thermal processing of powder particles in Ar atmosphere was concluded as a processing route for alumina foam manufacturing, and an explanation is the following: despite the high stability of the aluminum oxide shell around the aluminum particles the oxide layers are disrupted due to the thermal expansion difference between molten aluminum and the solid alumina shell (coefficient of thermal expansion for aluminum: 27.4 × 10^−6^ °C^−1^; for aluminum oxide: 7.4 × 10^−^^6^ °C^−1^) [48]. Thus, the thermal expansion mismatch generates sufficient stress to disrupt the alumina shells. As a consequence, aluminum particles melt together and leave behind less porosity as compared to samples thermally processed in air [49]. However, oxide crack healing appears simultaneously with the process of the oxide shell rupture if oxygen traces exist in a furnace. A fresh aluminum actively reacts with the oxygen in a thermal processing atmosphere, healing the cracks and forming new oxides into the place of cracks [50,51]. Therefore, it might be a reason why the thermally processed foams are still stable at 750–900 °C and do not melt completely. Despite the negative influence of the aluminum oxide layer on the homogeneity of strut structure, this oxide shells surrounding support the foam structure to survive the thermal treatment; that might be seen as an auxiliary skeleton in this system. This oxide network keeps the foam structure unchanged and is essential for the structural integrity of the foams and their stability [49,52].

The aluminum foams were prepared with a specific view to approximately the same total porosity in order to compare compressive strength and thermal properties of the foams with a porosity of 90–91% (Table 3). The total strut porosity included material pores, cavities (hollow strut pores) and cell pores. The cell porosity related to the foams without material pores. The total strut porosity consisted of the porosity of hollow struts and material pores. The strut porosity included closed and open pores. The hollow strut porosity related to the volume of the cavities after PU template burnout, which was calculated from the volumetric shrinkage of the foams.

The foams were characterized by shrinkage after thermal processing in the Ar atmosphere (Table 3), it is evident also from Figure 3. However, despite the linear shrinkage, the cell porosity of the thermally processed foams in Ar increased due to a decreasing material porosity (Figure 4). The powder particles were better densified and particularly merged in comparison with the foams thermally processed in air.

The thermal conductivity *λ_f_* of the foams at ~90%–91% porosity changed from 0.45 W·m^−1^K^−1^ to 2.98 W·m^−1^K^−1^ for the foams thermally processed in air at 750 °C and Ar at 800 °C respectively (Table 4). The thermal conductivity of the foams thermally processed at 850 and 900 °C were not possible to determine due to shrinkage and deformation of the foam parts during thermal processing.

For a comparison of the cell porosity and material porosity influence on the thermal conductivity of the obtained foams, Equations (1) and (2) were applied [42,53,54]. These equations are estimations for the conductivity of the porous strut material *λ_s_* (effect of the cell porosity) and the thermal conductivity of the bulk strut material without porosity *λ_b_* (effect of the material porosity) depending on a measured thermal conductivity of the foam *λ_f_*. A model derived by Ashby [53] is applied to calculate the thermal conductivity *λ_s_* of the porous strut material excluding the porosity within the strut (Equation (1)):

(1) $$\lambda_{s}=\frac{\lambda_{f}-P_{\mathrm{cell}}\cdot \lambda_{g}}{1/3\cdot \left(1-P_{\mathrm{cell}}\right)},$$

in this equation, *λ_g_* is the thermal conductivity of the gas phase (air) which is 0.0264 W·m^−1^K^−1^ [55], and *P_cell_* is the cell porosity from Table 3. The calculated strut thermal conductivity *λ_s_* is shown in Table 4. A model derived by Eucken (Equation (2)) was used for the calculation of the bulk thermal conductivity *λ_b_* of a porous material (in our case the thermal conductivity of the porous struts) [54]:

(2) $$\lambda_{s}=\lambda_{b}\frac{1+2P_{s}\cdot \left(1-\frac{\lambda_{g}}{\lambda_{b}}\right)/\left(2\frac{\lambda_{g}}{\lambda_{b}}+1\right)}{1-2P_{s}\cdot \left(1-\frac{\lambda_{g}}{\lambda_{b}}\right)/\left(2\frac{\lambda_{g}}{\lambda_{b}}+1\right)},$$

in this equation, *P_s_* is the strut porosity (material porosity) excluding the hollow strut cavities (Table 3), and *λ_s_* is the thermal conductivity of the porous strut material calculated using Equation (1).

It is known that the thermal conductivity of pure bulk aluminum is ~205 W·m^−1^K^−1^ [56]. Lower values of the calculated *λ_s_* in comparison with the bulk material is typical for porous materials in general. Therefore, *λ_s_* shows the influence of cell porosity on the thermal conductivity. The discrepancy between the thermal conductivity of pure bulk aluminum and *λ_b_* (Table 4) may be explained by impurities in the thermally processed aluminum powder [42,57,58], in our case the oxide shells around powder particles (alumina thermal conductivity is 24–39 W·m^−1^K^−1^ [59]). Consequently, low values of the measured *λ_f_* could be related not only to the open-cellular foam structure, but also the negative influence of oxide impurities in the strut material.

**Figure 6:**
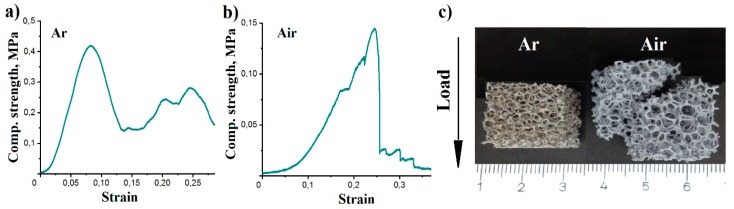
(**a**,**b**) Stress–strain curves and (**c**) foams after compressive strength test for a foam processed in Ar (left) and in Air (middle) for 3 h at 750 °C.

The compressive strength behavior and results are shown in Figure 6 and Table 5. The stress–strain curves of aluminum foams thermally processed for 3 h at 750 °C in air and Ar show a completely different behavior. For the foams thermally processed in air they point out a brittle behavior with shear fracture (Figure 6b,c) typical for ceramic foams. Foams processed in Ar possessed a more ductile behavior, which is typical for aluminum, or metals, in general (Figure 6a,c).

From the comparison of the aluminum foams sintered in air and Ar it follows that the compressive strength of the foams increased with a reduction of the aluminum oxide content and the total strut porosity (Table 5). The compressive strength of the foam thermally processed in the air reached 0.133 MPa, that for foams thermally processed in Ar at 750 °C was 0.339 MPa. Thermal processing in Ar improved the structural interconnectivity through the formation of molten aluminum agglomerations, which reduced the total strut porosity with the consequence of an increased compressive strength.

## 4. Conclusions

Open-cell aluminum foams were manufactured with a 20 ppi polyurethane template by the sponge replication technique. The open-cell green foams were thermally processed at 750 °C in air and in Ar at 750–900 °C. The total porosity of the thermally processed aluminum foams was between 90% and 91% and their microstructure showed typical hollow struts resulting from Schwartzwalder processing and porous struts resulting from incomplete thermal processing.

The thermal processing conditions and the processing atmosphere play a critical role in the microstructure formation, phase content, porosity, thermal conductivity, and mechanical properties. Open-cell aluminum foams thermally processed in Ar at temperature up to 800 °C possess a denser structure, a lower level of strut porosity, and a higher thermal conductivity and compressive strength. This effect, in comparison to foams thermally processed in air, was assigned to a lower amount of aluminum oxide after thermal processing resulting in thinner oxide shells around the starting aluminum particles and a steady disruption of the alumina shells during thermal processing. After this first study, further research is necessary to reduce the alumina/oxygen content in aluminum foams and to tailor their mechanical and thermal properties with a minimized second/alumina phase within the alumina foams.

## Figures and Tables

**Table 1:** Thermal processing conditions for binder and polyurethane (PU) burn out and thermal processing of the open-cell aluminum foams.

Binder Burning		PU Burning		Thermal Processing		
T, °C	Time, h	T, °C	Time, h	T, °C	Atmosphere	Time, h
250	3	500	3	750	Air	3
				750	Ar	
				800		
				850		
				900		

**Table 2:** Phase composition of the as-received powder and the aluminum foams thermally processed for 3 h at 750 °C in air and at 750–900 °C in Ar.

Sample	α-Al_2_O_3_, wt.%	γ-Al_2_O_3_, wt.%
Al powder	-	-
Air 750 °C	20.2	2.4
Ar 750 °C	-	4.6
Ar 800 °C	-	3.8
Ar 850 °C	1.5	4.3
Ar 900 °C (shrink part of the foam)	28.8	7.4

**Table 3:** Total porosity, open strut porosity, and compressive strength of aluminum foams thermally processed for 3 h at 750 °C in air and at 750–850 °C in Ar.

Sample	Linear Shrinkage, %	Total Porosity ^a^, %	Cell Porosity (P_cell_) ^b^, %	Total Strut Porosity ^c^, %	Strut Porosity (P_s_) ^d^, %	Hollow Strut Porosity ^e^, %
Air 750°C	0	90.4	78.6	58.5	51.6	6.9
Ar 750 °C	21	90.7	85.9	39.7	29.8	9.9
Ar 800 °C	21	90.2	86.6	35.6	25.0	10.6
Ar 850 °C	23	90.9	86.5	40.0	29.5	10.5

^a^ (V_material pores_+V_hollow strut pores_+V_cell pores_)/V_foam_; ^b^ Including the cavities after PU template burnout (V_cell pores_+V_hollow strut pores_)/V_foam_; ^c^ Related to the overall strut volume (V_hollow strut pores_+V_material pores_)/(V_material_+V_hollow strut pores_+V_material pores_); ^d^ Related to the strut volume V_material pores_/(V_hollow strut pores_+V_material_+V_material pores_); ^e^ The cavities after PU template burn out.

**Table 4:** Thermal conductivity of aluminum foams thermally processed at 750 °C in air and in Ar at 750–800 °C.

Sample	Therm. Cond. of Foam λ_f_, W·m^−1^K^−1^	Therm. Cond. of Porous Strut Material λ_s_, W·m^−1^K^−1^	Bulk Therm. Cond. of Bulk Material λ_b_, W·m^−1^ K^−1^
Air 750 °C	0.45 ± 0.05	6.0	17.1
Ar 750 °C	2.32 ± 0.14	38.4	66.8
Ar 800 °C	2.98 ± 0.17	66.0	103.0

**Table 5:** Total porosity, open strut porosity, and compressive strength of aluminum foams thermally processed for 3 h at 750 °C in air and at 750–850 °C in Ar.

Sample	Comp. Strength, MPa	Weibull Parameter m	Total Strut Porosity, %	Aluminum Oxides, wt.%
Air 750 °C	0.133 ± 0.023	7.2	58.5	22.6
Ar 750 °C	0.339 ± 0.078	3.2	39.7	4.6
Ar 800 °C	0.304 ± 0.051	6.56	36.5	3.8
Ar 850 °C	0.224 ± 0.030		40.0	5.8

## References

[1] Singh S., Bhatnagar N. (2018). A survey of fabrication and application of metallic foams (1925–2017). J. Porous Mater..

[2] Banhart J. (2001). Manufacture, characterisation and application of cellular metals and metal foams. Prog. Mater. Sci..

[3] Kim S., Lee C.-W. (2014). A Review on Manufacturing and Application of Open-cell Metal Foam. Procedia Mater. Sci..

[4] Bortolozzi J.P., Banús E.D., Milt V.G., Gutierrez L.B., Ulla M.A. (2010). The significance of passivation treatments on AISI 314 foam pieces to be used as substrates for catalytic applications. Appl. Surf. Sci..

[5] Gancarczyk A., Sindera K., Iwaniszyn M., Piątek M., Macek W., Jodłowski P.J., Wroński S., Sitarz M., Łojewska J., Kołodziej A. (2019). Metal Foams as Novel Catalyst Support in Environmental Processes. Catalysts.

[6] Li C.-L., Wang H., Zhou X., JieLi H.-z.L. (2010). Debinding of stainless steel foamprecursor with 3-D open-cell network structure. Trans. Nonfer. Met. Soc..

[7] Gupta N., Rohatgi P.K. (2014). Metal Matrix Syntactic Foams: Processing, Microstructure, Properties and Applications.

[8] Szlancsik A., Katona B., Károly D., Orbulov I.N. (2019). Notch (In)Sensitivity of Aluminum Matrix Syntactic Foams. Materials.

[9] Cybulski A., Moulijn J.A. (1994). Monoliths in Heterogeneous Catalysis. Catal. Rev..

[10] Hassani A., Habibolahzadeh A., Bafti H. (2012). Production of graded aluminum foams via powder space holder technique. Mater. Des..

[11] Parvanian A.M., Panjepour M. (2013). Mechanical behavior improvement of open-pore copper foams synthesized through space holder technique. Mater. Des..

[12] Choe H. (2004). Synthesis, structure, and mechanical properties of Ni–Al and Ni–Cr–Al superalloy foams. Acta Mater..

[13] Mutlu I., Oktay E. (2013). Mechanical properties of sinter-hardened Cr–Si–Ni–Mo based steel foam. Mater. Des..

[14] García-Moreno F. (2016). Commercial Applications of Metal Foams: Their Properties and Production. Materials.

[15] Quadbeck P., Kümmel K., Hauser R., Standke G., Adler J., Stephani G. Open Cell Metal Foams–Application-oriented Structure and Material Selection. Proceedings of the CELLMAT 2010.

[16] Soni B., Biswas S. (2015). Development of Al Foams by a Low-cost Salt Replication Method for Industrial Applications. Mat. Today Proc..

[17] Li Q., Bjerrum N.J. (2002). Aluminum as anode for energy storage and conversion: A review. J. Power Sources.

[18] Egan D.R., Ponce de León C., Wood R.J.K., Jones R.L., Stokes K.R., Walsh F.C. (2013). Developments in electrode materials and electrolytes for aluminium–air batteries. J. Power Sources.

[19] Liu Y., Sun Q., Li W., Adair K.R., Li J., Sun X. (2017). A comprehensive review on recent progress in aluminum–air batteries. Green Energy Environ..

[20] Quadbeck P., Kümmel K., Hauser R., Standke G., Adler J., Stephani G., Kieback B. (2011). Structural and Material Design of Open-Cell Powder Metallurgical Foams. Adv. Eng. Mater..

[21] Banhart J. (2003). Aluminum Foams: On the Road to Real Applications. MRS Bull..

[22] Zaragoza G., Goodall R. (2013). Metal Foams with Graded Pore Size for Heat Transfer Applications. Adv. Eng. Mater..

[23] Yang X., Hu Q., Du J., Song H., Zou T., Sha J., He C., Zhao N. (2019). Compression fatigue properties of open-cell aluminum foams fabricated by space-holder method. Int. J. Fatigue.

[24] Salvo L., Martin G., Suard M., Marmottant A., Dendievel R., Blandin J.-J. (2014). Processing and structures of solids foams. C. R. Phys..

[25] Davies G.J., Zhen S. (1983). Metallic foams: Their production, properties and applications. J. Mater. Sci..

[26] Kennedy A., Kondoh K. (2012). Porous Metals and Metal Foams Made from Powders. Powder Metallurgy.

[27] Karl S., Somers A.V. (1963). Method of Making Porous Ceramic Articles. U.S. Patent.

[28] Jamaludin A.R., Kasim S.R., Ismail A.K., Abdullah M.Z., Ahmad Z.A. (2015). The effect of sago as binder in the fabrication of alumina foam through the polymeric sponge replication technique. J. Eur. Ceram. Soc..

[29] Dietrich B., Schell G., Bucharsky E.C., Oberacker R., Hoffmann M.J., Schabel W., Kind M., Martin H. (2010). Determination of the thermal properties of ceramic sponges. Int. J. Heat Mass Transfer.

[30] Bakan H.I., Korkmaz K. (2015). Synthesis and properties of metal matrix composite foams based on austenitic stainless steels–titanium carbonitrides. Mater. Des..

[31] Choudhary A., Pratihar S.K., Agrawal A.K., Behera S.K. (2018). Macroporous SiOC Ceramics with Dense Struts by Positive Sponge Replication Technique. Adv. Eng. Mater..

[32] Kwon H., Park D.H., Park Y., Silvain J.F., Kawasaki A., Park Y. (2010). Spark plasma sintering behavior of pure aluminum depending on various sintering temperatures. Metals Mater. Int..

[33] Trunov M.A., Umbrajkar M.S., Schoenitz M., Mang J.T., Dreizin E.L. (2006). Oxidation and melting of aluminum nanopowders. J. Phys. Chem. B.

[34] Zheng Y., Chen L., Su Y., Tan J., Bao L., Lu Y., Wang J., Chen R., Chen S., Wu F. (2017). An interfacial framework for breaking through the Li-ion transport barrier of Li-rich layered cathode materials. J. Mater. Chem. A.

[35] Manonukul A., Srikudvien P., Tange M., Puncreobutr C. (2016). Geometry anisotropy and mechanical property isotropy in titanium foam fabricated by replica impregnation method. Mater. Sci. Eng. A.

[36] Li J.P., Li S.H., van Blitterswijk C.A., de Groot K. (2005). A novel porous Ti6Al4V: Characterization and cell attachment. J. Biomed. Mater. Res. A.

[37] Zaman E., Keleş Ö. (2014). Open Cell Aluminum Foams Produced by Polymer Impregnation Method. Acta Phys. Pol. A.

[38] Coelho A.A. (2012). Topas Academic V5.

[39] Henon J., Alzina A., Absi J., Smith D.S., Rossignol S. (2013). Potassium geopolymer foams made with silica fume pore forming agent for thermal insulation. J. Porous Mater..

[40] Ross R.B. (1992). Metallic Materials Specification Handbook.

[41] (1993). Hochleistungskeramik; Monolithische Keramik; Allgemeine und strukturelle Eigenschaften; Teil 2: Bestimmung von Dichte und Porosität.

[42] Betke U., Lieb A., Scheffler F., Scheffler M. (2017). Manufacturing of Reticulated Open-Cellular Aluminum Nitride Ceramic Foams from Aqueous AlN Suspensions. Adv. Eng. Mater..

[43] Log T., Gustafsson S.E. (1995). Transient plane source (TPS) technique for measuring thermal transport properties of building materials. Fire Mater..

[44] He Y. (2005). Rapid thermal conductivity measurement with a hot disk sensor. Thermochim. Acta.

[45] Sercombe T.B., Schaffer G.B. (2003). Rapid manufacturing of aluminum components. Science.

[46] Olakanmi E.O., Cochrane R.F., Dalgarno K.W. (2015). A review on selective laser sintering/melting (SLS/SLM) of aluminium alloy powders: Processing, microstructure, and properties. Prog. Mater. Sci..

[47] Patnaik P. (2002). Handbook of Inorganic Chemicals.

[48] Harper C.A. (2001). Handbook of Ceramics, Glasses and Diamond.

[49] Barg S., Soltmann C., Schwab A., Koch D., Schwieger W., Grathwohl G. (2011). Novel open cell aluminum foams and their use as reactive support for zeolite crystallization. J. Porous Mater..

[50] Hasani S., Panjepour M., Shamanian M. (2012). Oxidation and Kinetic Analysis of Pure Aluminum Powder under Nonisothermal Condition. J. Aquac. Res. Dev..

[51] Liu Y., Ren H., Jiao Q.J. (2017). Oxidation mechanism of micron-sized aluminum particles in Al-CO 2 gradually heating system. IOP Conf. Ser. Mater. Sci. Eng..

[52] Körner C., Arnold M., Singer R.F. (2005). Metal foam stabilization by oxide network particles. Mater. Sci. Eng. A.

[53] Ashby M.F. (2006). The properties of foams and lattices. Philos. Trans. A Math. Phys. Eng. Sci..

[54] Eucken A. (1932). Die Wärmeleitfähigkeit feuerfester Stoffe. Ihre Berechnung aus der Wärmeleitfähigkeit der Bestandteile.

[55] Lemmon E.W., Jacobsen R.T. (2004). Viscosity and Thermal Conductivity Equations for Nitrogen, Oxygen, Argon, and Air. Int. J. Thermophys..

[56] Touloukian Y.S., Powell R.W., Ho C.Y., Klemens P.G. (1973). Thermophysical Properties of Matter—The TPRC Data Series.

[57] McNeil L.E., Grimsditch M., French R.H. (1993). Vibrational spectroscopy of aluminum nitride. J. Am. Ceram. Soc..

[58] Slack G.A., Tanzilli R.A., Pohl R.O., Vandersande J.W. (1987). The intrinsic thermal conductivity of AIN. J. Phys. Chem. Solids.

[59] Smith D.S., Fayette S., Grandjean S., Martin C., Telle R., Tonnessen T. (2003). Thermal resistance of grain boundaries in alumina ceramics and refractories. J. Am. Ceram. Soc..

